# Mortalidade em Cinco Anos em Sobreviventes de Cirurgia de Revascularização do Miocárdio: Um Estudo Retrospectivo de um Centro Terciário

**DOI:** 10.36660/abc.20250362

**Published:** 2026-05-06

**Authors:** Eduardo Martelli Moreira, Bernadette Cunha Waldvogel, Eduardo Bello Martins, Desiderio Favarato, Eduardo Gomes Lima, Fabio Grunspun Pitta, Fabiana Hanna Rached, Henrique Trombini Pinesi, Omar Asdrúbal Vilca Mejia, Carlos Vicente Serrano

**Affiliations:** 1 Instituto do Coração do Hospital das Clínicas Faculdade de Medicina Universidade de São Paulo São Paulo SP Brasil Instituto do Coração do Hospital das Clínicas da Faculdade de Medicina da Universidade de São Paulo, São Paulo, SP – Brasil; 2 Universidade Federal do Paraná Curitiba PR Brasil Universidade Federal do Paraná, Curitiba, PR – Brasil; 3 Fundação Sistema Estadual de Análise de Dados São Paulo SP Brasil Fundação Sistema Estadual de Análise de Dados São Paulo, SP – Brasil; 4 Hospital Israelita Albert Einstein São Paulo SP Brasil Hospital Israelita Albert Einstein, São Paulo, SP – Brasil

**Keywords:** Angina Pectoris, Revascularização Miocárdica, Epidemiologia Descritiva

## Abstract

**Fundamento:**

Dados sobre desfechos em longo prazo após cirurgia de revascularização do miocárdio (CRM) no Brasil ainda são limitados, embora essenciais para orientar políticas de saúde pública e a prática clínica.

**Objetivos:**

Estimar as taxas de mortalidade por todas as causas e por causas específicas em cinco anos em pacientes submetidos à CRM e identificar fatores de risco associados.

**Métodos:**

Análise retrospectiva de 6933 pacientes submetidos à CRM no Instituto do Coração, Faculdade de Medicina da Universidade de São Paulo, entre 2007 e 2018. Os dados de mortalidade até 2018 foram obtidos em bases de dados governamentais. Adotou-se nível de significância de 5%.

**Resultados:**

A coorte do estudo incluiu 4854 homens (72%), com idade mediana de 63 anos (IIQ: 57–70). Diabetes mellitus estava presente em 2.984 pacientes (43%), e 1.918 (29%) tinham doença renal crônica. Durante o seguimento, ocorreram 1075 óbitos, dos quais 506 (47,1%) foram atribuídos a causas cardiovasculares. A taxa de mortalidade por todas as causas em 5 anos foi de 12,9% (IC 95%: 11,9–13,9), enquanto a mortalidade cardiovascular foi de 5,7% (IC 95%: 5,1–6,4). A principal causa de morte cardiovascular foi doença isquêmica do coração (290 óbitos, 57,3%), seguida por doença cerebrovascular (84 óbitos, 16,6%). Fatores significativamente associados à mortalidade por todas as causas incluíram idade avançada, diabetes mellitus, taxa de filtração glomerular, HDL-colesterol, fração de ejeção do ventrículo esquerdo, dimensões do átrio esquerdo e do ventrículo esquerdo em diástole, internação de urgência, cirurgia com circulação extracorpórea e tempo de internação hospitalar.

**Conclusão:**

Pacientes submetidos à cirurgia de revascularização do miocárdio em um centro terciário apresentaram taxas moderadas de mortalidade por todas as causas e cardiovascular em cinco anos. Esses achados destacam fatores prognósticos importantes que podem auxiliar na estratificação de risco e orientar estratégias de manejo pós-operatório.

## Graphical abstract

**Figura Central:**
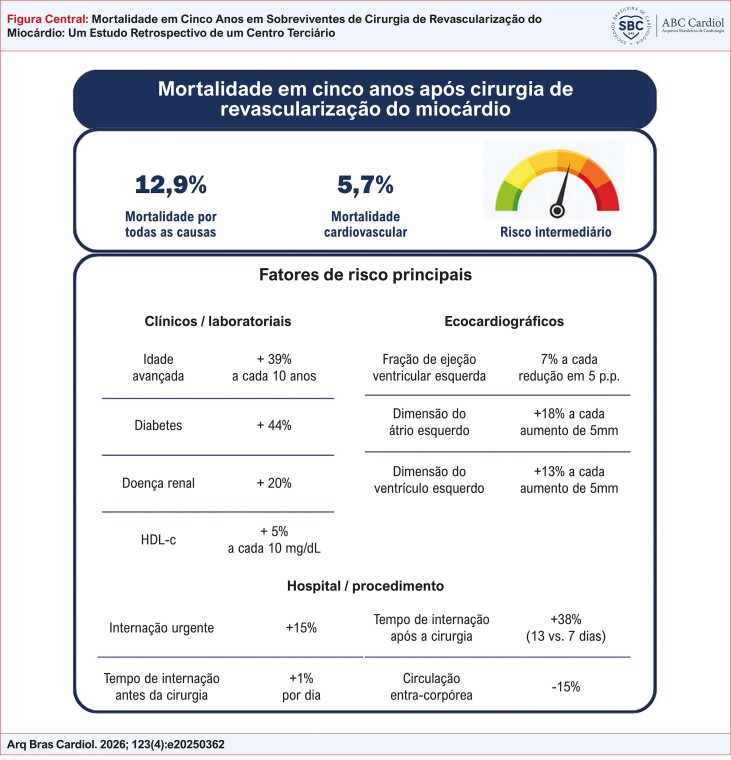
: Mortalidade em Cinco Anos em Sobreviventes de Cirurgia de Revascularização do Miocárdio: Um Estudo Retrospectivo de um Centro Terciário Principais resultados.

## Introdução

A doença arterial coronariana (DAC) permanece como a principal causa de morbidade e mortalidade em todo o mundo, particularmente entre indivíduos com mais de 30 anos de idade.^1^ Embora registros multinacionais evidenciem diferenças relevantes nos perfis clínicos e nos desfechos, países da América Central e do Sul relatam consistentemente maiores taxas de diabetes mellitus (DM), hipertensão, infarto do miocárdio e mortalidade cardiovascular.^2-4^ Apesar dessa expressiva carga de doença, essas regiões são frequentemente sub-representadas em estudos de grande escala.

No Brasil, as doenças cardiovasculares respondem por um terço de todos os óbitos, sendo a DAC responsável por quase metade dessas fatalidades.^5^ Embora iniciativas conduzidas por sociedades nacionais de cardiologia e cirurgia cardiovascular busquem aprimorar o cuidado, ainda há escassez de dados sobre os desfechos em longo prazo desses pacientes.^6-8^

Este estudo busca preencher essa lacuna ao avaliar a mortalidade por todas as causas e por causas específicas em cinco anos em uma coorte de sobreviventes de cirurgia de revascularização do miocárdio (CRM). Além disso, pretende identificar os principais fatores de risco associados a esses desfechos.

## Métodos

Este estudo foi aprovado pelo Comitê de Ética do hospital (protocolo nº 4.513.250), que dispensou a exigência de consentimento individual dos pacientes. Devido a restrições legais, os dados não podem ser disponibilizados publicamente. O estudo segue as diretrizes do *Strengthening the Reporting of Observational Studies in Epidemiology* (STROBE).^9^

### Pacientes e cenário

Este estudo de coorte retrospectivo foi conduzido no Instituto do Coração (Faculdade de Medicina da Universidade de São Paulo), Brasil. Foram incluídos pacientes submetidos à CRM entre 2007 e 2018 que sobreviveram à hospitalização inicial. Apenas o primeiro procedimento de CRM foi considerado para inclusão. Foram excluídos pacientes residentes fora do Estado de São Paulo, menores de 18 anos, aqueles submetidos a procedimentos combinados valvares ou da aorta, e os que faleceram durante a hospitalização.

### Fontes de dados e definições

Os dados clínicos foram coletados a partir de prontuários eletrônicos, incluindo exames laboratoriais, ecocardiografia, uso de medicamentos, tempo de internação e detalhes cirúrgicos. A taxa de filtração glomerular estimada (TFGe) foi calculada de acordo com o método da *Chronic Kidney Disease Epidemiology Collaboration* de 2021, e a doença renal crônica foi definida como TFGe ≤ 60 mL/min/1,73 m^2^.^10^ O DM foi definido como o uso de medicamentos antidiabéticos, hemoglobina glicada > 6,5% ou glicemia de jejum > 126 mg/dL.^1 1^ A hipertensão foi definida como a prescrição de inibidores da enzima conversora de angiotensina, bloqueadores dos receptores de angiotensina, bloqueadores dos canais de cálcio, clonidina ou diuréticos.

Os dados de mortalidade, incluindo a causa de óbito codificada pela Classificação Internacional de Doenças, foram obtidos da Fundação Sistema Estadual de Análise de Dados, um banco de dados de agência governamental. Essa agência é responsável pelo registro civil do Estado de São Paulo.^1 2^ Como não havia informações disponíveis para pacientes residentes em outros estados, eles foram excluídos deste relatório.

Os dados dos prontuários eletrônicos e do banco de dados governamental de mortalidade foram vinculados utilizando identificadores dos pacientes, como nome, nome da mãe, número de identidade e data de nascimento, de acordo com critérios de pareamento pré-especificados. Esses critérios incluíram tanto correspondências exatas quanto parciais. Todos os pares correspondentes (um registro de cada base de dados) foram validados por dois investigadores independentes. Um par foi considerado válido se ambos os investigadores concordassem; discrepâncias foram resolvidas por consenso. A confidencialidade dos dados foi assegurada pela realização da vinculação in loco na agência governamental, com acesso aos registros identificados restrito apenas aos dois investigadores.

### Acompanhamento e desfechos

O acompanhamento iniciou-se no dia da alta hospitalar e terminou no momento do óbito, conforme registrado nos atestados de óbito. Pacientes sem registro de óbito foram considerados vivos, e seu acompanhamento foi censurado na última data de contato com o hospital (como consulta ambulatorial, exame laboratorial ou ecocardiografia), ou em 31 de dezembro de 2018. Como um importante centro terciário no Brasil, nossa instituição fornece acompanhamento contínuo para a maioria dos pacientes cirúrgicos. A mortalidade cardiovascular foi definida pelos códigos do capítulo I da Classificação Internacional de Doenças.

### Análise estatística

As variáveis categóricas foram apresentadas como números absolutos e porcentagens, enquanto as variáveis contínuas foram expressas como medianas com intervalos interquartis (IIQs). As variáveis contínuas foram avaliadas quanto à normalidade por meio de análise gráfica e do teste de Kolmogorov-Smirnov. Dada a natureza retrospectiva deste estudo, não foram realizados cálculos de tamanho amostral; em vez disso, todos os participantes elegíveis foram incluídos. As comparações de linha de base com os participantes excluídos foram conduzidas utilizando o teste de soma de postos de Wilcoxon e o teste do qui-quadrado, conforme apropriado. A mortalidade por todas as causas foi estimada pelo método de Kaplan–Meier, enquanto a mortalidade por causas específicas foi avaliada pela abordagem de incidência cumulativa.

Os fatores de risco foram avaliados por meio de um modelo de riscos proporcionais de Cox. Inicialmente, todas as variáveis eram elegíveis para inclusão. Variáveis identificadas como altamente correlacionadas — com base em conhecimento prévio, correlações de Spearman e Pearson — foram excluídas. Variáveis categóricas com prevalência muito baixa ou muito alta (<5% ou >95%) também foram excluídas devido a considerações de poder estatístico. As variáveis contínuas foram avaliadas quanto à não linearidade por meio de resíduos de Martingale e análise gráfica de modelos preliminares. Variáveis não lineares foram modeladas como *splines* cúbicas restritas (nós nos percentis 10, 50 e 90). Os resíduos de Schoenfeld foram utilizados para testar a suposição de riscos proporcionais. Todas as variáveis foram inicialmente analisadas em modelos não ajustados e, posteriormente, em modelos totalmente ajustados.

As análises estatísticas foram realizadas com o software R, versão 4.5.0.^1 3^ Todas as análises foram consideradas exploratórias, e não foram feitos ajustes para múltiplos testes. Adotou-se um nível de significância de 5%. Os resultados são apresentados como valores de p, razões de risco (HR) e intervalos de confiança de 95% (IC95%).

### Tratamento de dados faltantes

As estimativas de mortalidade foram realizadas com base em casos completos. Para a análise de fatores de risco, os dados foram considerados como ausentes ao acaso. Portanto, foi realizada imputação múltipla utilizando o algoritmo de *predictive mean matching* com *bootstrapping* com reposição, conforme implementado pela função *aregImpute* do pacote Hmisc (versão 5.1-1).^14^

## Resultados

**Figura 1:**
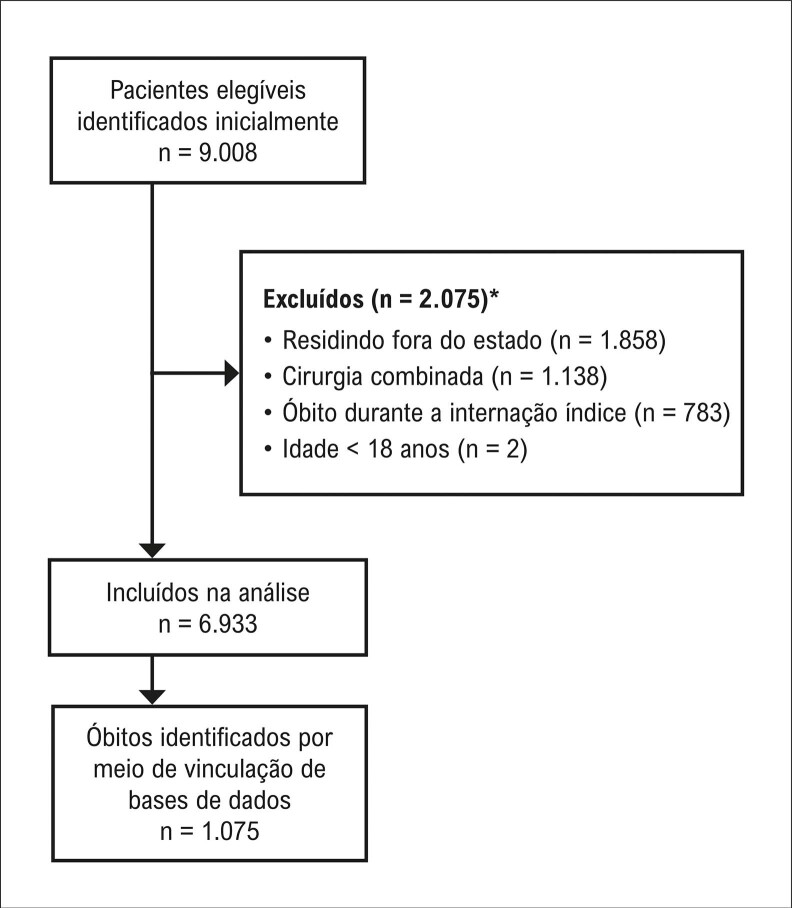
– Fluxograma do estudo. *Pacientes poderiam preencher mais de um critério.

Inicialmente, um total de 9008 pacientes elegíveis submetidos à CRM entre 2007 e 2018 foram identificados. Desses, 2075 pacientes foram excluídos por diversos motivos (Figura 1), resultando em 6.933 pacientes incluídos na análise. Dentre estes, foram recuperados 1075 atestados de óbito.

Comparados aos pacientes excluídos, os incluídos apresentaram maior prevalência de homens (72% vs. 68%, p < 0,001), menor prevalência de doença renal crônica (29% vs. 39%, p < 0,002), eram mais jovens (idade mediana de 63 vs. 67 anos, p < 0,001) e tinham maior fração de ejeção ventricular esquerda mediana (60% vs. 57%, p < 0,001). Outras características da amostra estão descritas na Tabela 1. O período total de acompanhamento foi de 31610 pacientes-ano (mediana de 4,0 anos; IIQ 1,4-7,3).

**Tabela 1:** – Características clínicas, ecocardiográficas e cirúrgicas basais dos pacientes (n=6.933)

	**Amostra total (N = 6933)**	**Óbito durante o acompanhamento**		**Dados faltantes**
		**Número (N = 5858)**	**Sim (N = 1075)**	
**Homens, n (%)**	4.854 (72)	4.142 (73)	712 (69)	237 (3.4%)
**Idade, anos, Mediana (IIQ)**	63 (57-70)	63 (56-69)	67 (59-73)	0 (0%)
**Doença renal crônica, n (%)**	1.918 (29)	1.465 (26)	453 (44)	237 (3.4%)
**Diabetes mellitus, n (%)**				**0 (0%)**
Dependentes de insulina	1.440 (21)	1.149 (20)	291 (27)	
Não dependentes de insulina	1.544 (22)	1.310 (22)	234 (22)	
Sem DM	3.949 (57)	3.399 (58)	550 (51)	
**Hipertensão, n (%)**	4.742 (69)	3.912 (67)	830 (78)	24 (0.3%)
**Colesterol total, mg/dL, Mediana (IIQ)**	164 (137-197)	164 (137-197)	164 (136-196)	1.107 (16%)
**LDL-colesterol, mg/dL, Mediana (IIQ)**	95 (73-124)	95 (72-124)	97 (75-125)	1.318 (19%)
**HDL-colesterol, mg/dL, Mediana (IIQ)**	37 (31-44)	37 (31-44)	36 (31-44)	1.138 (16%)
**Triglicerídeos, mg/dL, Mediana (IIQ)**	131 (94-185)	132 (95-186)	128 (91-182)	1.107 (16%)
**Creatinina, mg/dL, Mediana (IIQ)**	1.07 (0.91-1.29)	1.06 (0.91-1.26)	1.17 (0.94-1.49)	0 (0%)
**Taxa de Filtração Glomerular, mL/min/1,73 m^2^, Mediana (IIQ)**	73 (57-88)	74 (59-90)	64 (46-83)	237 (3.4%)
**FEVE, %, Mediana (IIQ)**	60 (51-65)	61 (53-65)	57 (42-63)	2.046 (30%)
**Diâmetro diastólico do VE, mm, Mediana (IIQ)**	50 (46-54)	50 (46-53)	51 (47-56)	2.155 (31%)
**Tipo de enxerto da artéria torácica interna (%)**				1.091 (16%)
Duplo	680 (12)	643 (13)	37 (4.9)	
Simples	4.783 (82)	4.154 (82)	629 (83)	
Sem enxerto da artéria torácica interna	379 (6.5)	287 (5.6)	92 (12)	
**Enxerto de veia safena (%)**	5.221 (89)	4.531 (89)	690 (91)	1.091 (16%)
**Padrão de seleção dos enxertos, n (%)**				**1.091 (16%)**
Arterial	621 (11)	553 (11)	68 (9.0)	
Misto	4.852 (83)	4.252 (84)	600 (79)	
Venoso	369 (6.3)	279 (5.5)	90 (12)	
**Territórios enxertados, n (%)**				**1.091 (16%)**
Um	617 (11)	530 (10)	87 (11)	
Dois	2.601 (45)	2.245 (44)	356 (47)	
Três	2.624 (45)	2.309 (45)	315 (42)	
**Enxerto para ADAE, n (%)**	5.742 (98)	5.006 (98)	736 (97)	1.091 (16%)
**Enxerto para ACD, n (%)**	3.580 (61)	3.148 (62)	432 (57)	1.091 (16%)
**Enxerto para ACX, n (%)**	4.369 (75)	3.793 (75)	576 (76)	1.091 (16%)
**Cirurgia com circulação extracorpórea, n (%)**	5.978 (86)	5.139 (88)	839 (78)	3 (<0,1%)
**Admissão na urgência, n (%)**	3.144 (45)	2.594 (44)	550 (51)	3 (<0,1%)
**Dias de internação antes da cirurgia, Mediana (IIQ)**	5,7 (1,8-8,7)	5,5 (1,4-8,2)	7,0 (3,6-12,0)	56 (0,8%)
**Dias de internação após a cirurgia, Mediana (IIQ)**	9 (7-13)	8 (7-12)	11 (8-19)	56 (0,8%)
**Ano da cirurgia, Mediana (IIQ)**	2012 (2009-2015)	2013 (2010-2016)	2009 (2008-2012)	0 (0%)

FEVE: fração de ejeção do ventrículo esquerdo; II: intervalo interquartil; VE: ventrículo esquerdo; ADAE: artéria descendente anterior esquerda; ACD: artéria coronária direita; ACX: artéria circunflexa cirurgia com circulação extracorpórea.

### Mortalidade por todas as causas e por causas específicas

**Figura 2:**
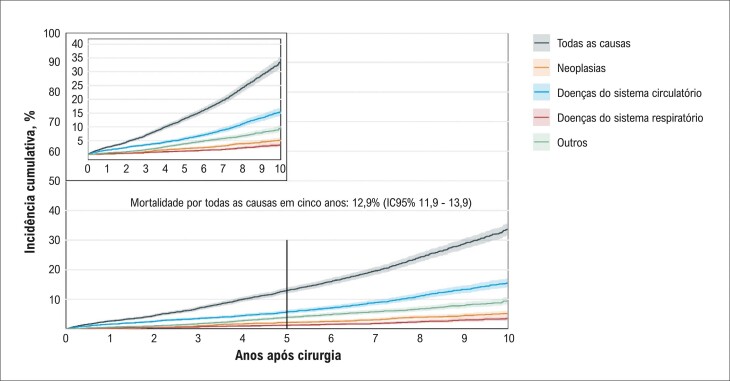
– Óbitos cumulativos por todas as causas e por causa específica.

Durante o período do estudo, foram registrados 1075 óbitos (Figura 2). A taxa de mortalidade por todas as causas em cinco anos foi de 12,9% (IC 95%: 11,9–13,9), sendo que os óbitos cardiovasculares representaram 5,7% (IC 95%: 5,1–6,4) dos casos (Figura Central). A doença cardiovascular foi a principal causa de morte, seguida por neoplasias malignas e doenças respiratórias (Tabela 2).

**Tabela 2:** – Óbitos por todas as causas e por causas específicas, de acordo com o tempo de seguimento

	**n**	**%**	**Incidência no ano de seguimento, % (IC95%)**		
**Causa da morte**			**1**	**5**	**10**
Todas as causas			2,7 (2,3-3,1)	12,9 (11,9-13,9)	33,7 (31,6-35,7)
Doenças do sistema circulatório	506	47,1	1,6 (1,3-2,0)	5,7 (5,1-6,4)	15,6 (14,1-17,2)
Neoplasias	163	15,2	0,2 (0,11-0,35)	2,1 (1,75-2,61)	5,2 (4,36-6,18)
Doenças do sistema respiratório	108	10,0	0,29 (0,18-0,45)	1,23 (0,95-1,59)	3,50 (2,81-4,36)
Outros	298	27,7	0,57 (0,41-0,79)	3,85 (3,32-4,46)	9,42 (8,29-10,71)

Mais da metade dos óbitos cardiovasculares (290 casos, 57,3%) foi atribuída à DAC, seguida por doença cerebrovascular (84 casos, 16,6%). As neoplasias malignas ocorreram principalmente no sistema digestivo (57 casos, 35%), sistema respiratório (31 casos, 19%) e neoplasias genitais ou mamárias (26 casos, 16%).

### Fatores de risco associados à mortalidade

**Figura 3:**
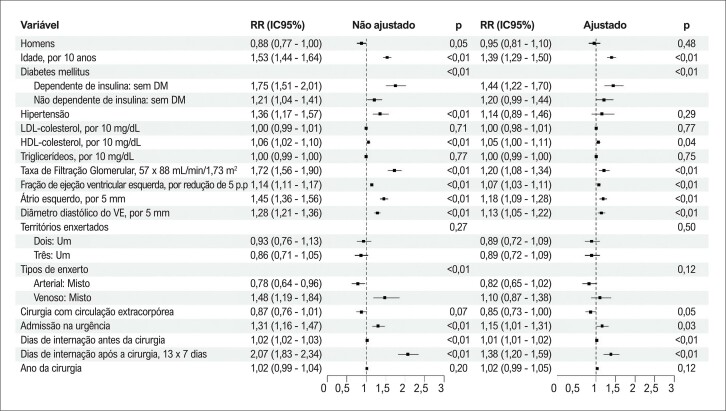
– Fatores associados com mortalidade por todas as causas.

As análises ajustadas e não ajustadas dos fatores de risco associados à mortalidade por todas as causas são apresentadas na Figura 3. Após o ajuste multivariado, idade, diabetes (particularmente dependente de insulina), HDL-colesterol, TFGe, fração de ejeção do ventrículo esquerdo, aumento do átrio esquerdo, diâmetro diastólico do ventrículo esquerdo, cirurgia com circulação extracorpórea, admissão hospitalar de urgência e os dias de hospitalização (tanto antes como após o procedimento) foram todos independentemente associados à mortalidade.

**Figura 4:**
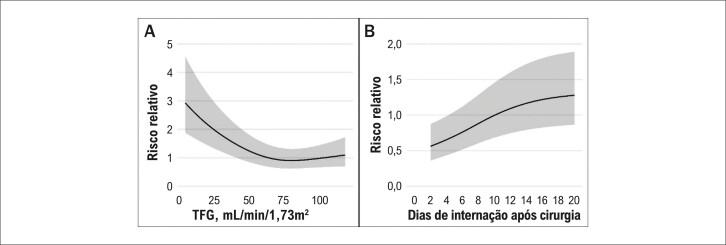
– Associação não linear entre (A) a taxa de filtração glomerular estimada e (B) os dias de internação após a cirurgia, e o risco de óbito.

A TFGe apresentou uma associação não linear com o desfecho: indivíduos com TFGe de 57 mL/min/1,73 m^2^ (3º quartil), em comparação com aqueles com 88 mL/min/1,73 m^2^ (1º quartil), tiveram HR de 1,20 (IC 95% 1,08–1,34; p < 0,01). O risco permaneceu estável acima de 60 mL/min/1,73 m^2^, mas aumentou substancialmente abaixo desse limiar (Figura 4).

Da mesma forma, os dias de hospitalização pós-procedimento também demonstraram uma associação não linear com a mortalidade. O risco aumentou rapidamente e atingiu um platô por volta do 16º dia de internação pós-procedimento (Figura 4). Em comparação com o 1º quartil (7 dias), indivíduos no 3º quartil (13 dias) apresentaram HR de 1,38 (IC 95% 1,20–1,59; p < 0,01).

**Figura 5:**
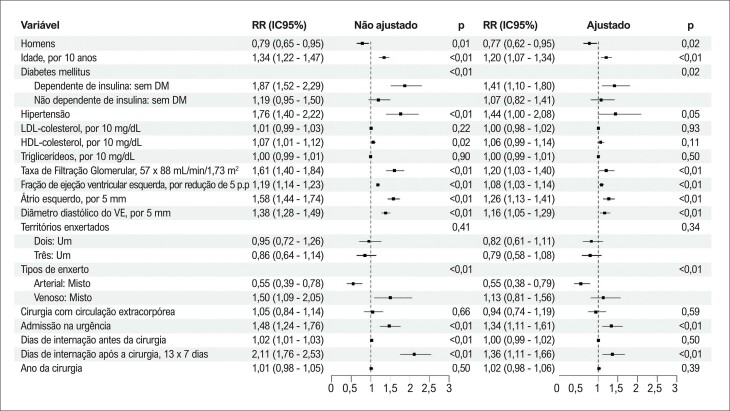
– Fatores associados com morte cardiovascular.

A morte cardiovascular esteve associada, em grande parte, aos mesmos fatores de risco, com algumas exceções (Figura 5). HDL-colesterol e os dias de hospitalização pré-procedimento não apresentaram associação com o desfecho (ambos p > 0,05). Em contraste, sexo masculino (HR 0,77; IC 95% 0,62–0,95; p = 0,02), revascularização exclusivamente com enxertos arteriais (HR 0,55 vs. tipos de enxertos mistos; IC 95% 0,38–0,79; p < 0,01) e hipertensão (HR 1,44; IC 95% 1,00–2,08; p = 0,05) foram significativamente associados à mortalidade cardiovascular.

## Discussão

### Achados principais

Nesta coorte de sobreviventes de CRM, a taxa de mortalidade por todas as causas em cinco anos foi de 12,9% (IC 95% 11,9–13,9), sendo a doença cardiovascular a principal causa de óbito, responsável por 5,7% (IC 95% 5,1–6,4) dos casos. Notavelmente, a própria DAC foi responsável por 57,3% dessas mortes cardiovasculares. Diversos fatores — incluindo idade, diabetes, níveis mais elevados de HDL-colesterol, menor TFGe, aumento das dimensões atrial e ventricular esquerdas, menor fração de ejeção do ventrículo esquerdo, cirurgia sem circulação extracorpórea, hospitalização de urgência e internação prolongada — foram associados ao aumento da mortalidade. Sexo masculino, revascularização exclusivamente com enxertos arteriais e hipertensão foram especificamente associados à mortalidade cardiovascular.

### Prognóstico

Embora este seja um estudo de centro único, nossa coorte é comparável ao registro da Sociedade Brasileira de Cirurgia Cardiovascular, um estudo multicêntrico conduzido em 17 instituições distribuídas por quatro das cinco regiões geográficas do Brasil. Nesse registro, foram incluídos 2.929 pacientes, com idade mediana de 63,5 anos; 29% eram mulheres; diabetes estava presente em 42,5% e hipertensão em 84,5%.^6^ Infelizmente, dados de seguimento em longo prazo dessa coorte não foram publicados.

De modo geral, encontramos taxas de mortalidade consistentes com aquelas relatadas na literatura internacional. Por exemplo, o Registro do Estado de Nova York, incluindo 8.597 pacientes, relatou uma mortalidade em 5 anos de 17,6%, enquanto dados do STS *Adult Cardiac Surgery Database* mostraram taxas de 8,1% em um ano e 23,3% em três anos.^15,16^ Um registro islandês documentou uma taxa de mortalidade em 5 anos de 10,1%, incluindo eventos cirúrgicos.^17^ Além disso, ensaios clínicos randomizados relataram taxas de mortalidade em 5 anos variando de 10% a 15% nos grupos submetidos à CRM, embora esses valores incluam mortalidade perioperatória.^1^

Considerando que as diretrizes internacionais definem alto risco cardiovascular como uma taxa anual de mortalidade cardiovascular superior a 3%, nossa população seria classificada como de risco moderado.^18-24^

### Fatores de risco associados

Idade, diabetes e TFGe foram consistentemente associados tanto à mortalidade por todas as causas quanto à mortalidade cardiovascular, como esperado. De forma interessante, TFGe e os dias de hospitalização pós-operatória apresentaram uma relação não linear com o risco. Enquanto o risco de mortalidade não se alterou de forma substancial para valores de TFGe acima de 60 mL/min/1,73 m^2^, ele aumentou de maneira acentuada abaixo desse limiar. Esse achado está alinhado com estudos prévios em coortes pós-CRM e na população geral.^25,26^

Apesar de tradicionalmente estar associado a um prognóstico favorável, o HDL-colesterol mostrou associação com mortalidade por todas as causas (mas não com mortalidade cardiovascular). Embora isso provavelmente represente um erro tipo I (falso positivo), vale destacar que um grande estudo populacional descreveu uma relação em formato de U entre HDL-colesterol e mortalidade.^27^

Marcadores ecocardiográficos de dano estrutural do miocárdio, como o diâmetro do ventrículo esquerdo e a fração de ejeção, foram previsivelmente associados tanto à mortalidade por todas as causas quanto à mortalidade cardiovascular.^28^ Além disso, o aumento do átrio esquerdo também se associou à mortalidade. Como a insuficiência cardíaca — independentemente da fração de ejeção — está intimamente relacionada à DAC, o aumento do átrio esquerdo pode refletir doença mais extensa e pressões de enchimento elevadas.^29,30^ Ademais, o aumento do átrio esquerdo está relacionado à fibrilação atrial, e já foi demonstrado que a fibrilação atrial incidente está associada a AVC e mortalidade após a cirurgia.^3 1^

Nossos achados sugerem que o tipo de revascularização (totalmente arterial, totalmente venosa ou mista) esteve associado à mortalidade cardiovascular, mas não à mortalidade por todas as causas. Ensaios clínicos randomizados têm mostrado resultados um tanto inconsistentes: enquanto enxertos bilaterais da artéria torácica interna não demonstraram superioridade em relação ao enxerto único, enxertos da artéria radial têm sido associados a melhores desfechos quando comparados aos enxertos de veia safena.^32,33^ Estudos observacionais, de modo geral, indicam um benefício da revascularização totalmente arterial.^34,35^

A eficácia das cirurgias com circulação extracorpórea (*on-pump*) versus sem circulação extracorpórea (*off-pump*) tem sido amplamente debatida. Estudos não randomizados, incluindo o nosso, sugerem potenciais benefícios de sobrevivência em longo prazo com procedimentos *on-pump*; no entanto, esses achados não têm sido consistentemente reproduzidos em ensaios clínicos randomizados.^36^

### Limitações

Este estudo apresenta várias limitações. Primeiro, todas as análises foram exploratórias e devem ser interpretadas principalmente como geradoras de hipóteses. Segundo, o uso de dados secundários limitou o acesso a algumas variáveis importantes, como anatomia coronariana, isquemia miocárdica, sintomas e medidas de qualidade de vida. Terceiro, nosso acesso às declarações de óbito ficou restrito ao Estado de São Paulo, o que pode ter resultado na exclusão de mortes ocorridas fora da região. Para mitigar esse problema, incluímos apenas pacientes residentes em São Paulo e acompanhados ativamente no Instituto do Coração.

## Conclusões

Os pacientes submetidos à CRM nesta coorte brasileira apresentaram taxas moderadas de mortalidade em cinco anos, de acordo com os padrões internacionais, sendo quase metade desses óbitos atribuída a causas cardiovasculares. Os principais fatores de risco associados à mortalidade incluíram idade, DM, TFGe, cirurgia sem circulação extracorpórea, anormalidades estruturais cardíacas, urgência da internação e duração da permanência hospitalar.
