# Supplementary material for: Mortalidade em Cinco Anos em Sobreviventes de Cirurgia de Revascularização do Miocárdio: Um Estudo Retrospectivo de um Centro Terciário
Source: Arq Bras Cardiol. 2026 May 6;123(4):e20250362. [Article in Portuguese] doi: 10.36660/abc.20250362 (PMC13400000; doi:10.36660/abc.20250362)
Supplement: Translation

# Translation: Five-Year Mortality among Survivors of Coronary Artery Bypass Graft Surgery: A Retrospective Study from a Tertiary Center

**DOI:** 10.36660/abc.20250362i

**Authors:** Eduardo Martelli Moreira, Bernadette Cunha Waldvogel, Eduardo Bello Martins, Desiderio Favarato, Eduardo Gomes Lima, Fabio Grunspun Pitta, Fabiana Hanna Rached, Henrique Trombini Pinesi, Omar Asdrúbal Vilca Mejia, Carlos Vicente Serrano

**Affiliations:** 1 Brazil Instituto do Coração do Hospital das Clínicas da Faculdade de Medicina da Universidade de São Paulo, São Paulo, SP – Brazil; 2 Brazil Universidade Federal do Paraná, Curitiba, PR – Brazil; 3 Brazil Fundação Sistema Estadual de Análise de Dados São Paulo, SP – Brazil; 4 Brazil Hospital Israelita Albert Einstein, São Paulo, SP – Brazil

**Keywords:** Angina Pectoris, Myocardial Revascularization, Descriptive Epidemiology

## Abstract

**Background:**

Long-term outcome data following Coronary Artery Bypass Graft (CABG) surgery in Brazil remain limited, yet are essential for informing public health policies and guiding clinical practice.

**Objectives:**

To estimate five-year all-cause and cause-specific mortality rates in patients undergoing CABG and to identify associated risk factors.

**Methods:**

A retrospective analysis of 6,933 patients who underwent CABG at the Heart Institute, Faculty of Medicine, University of São Paulo, between 2007 and 2018. Mortality data up to 2018 were retrieved from government databases. A significance level of 5% was adopted.

**Results:**

The study cohort included 4,854 men (72%) with a median age of 63 years (IQR: 57–70). Diabetes mellitus was present in 2,984 patients (43%), and 1,918 (29%) had chronic kidney disease. During follow-up, 1,075 deaths occurred, of which 506 (47.1%) were attributed to cardiovascular causes. The 5-year all-cause mortality rate was 12.9% (95% CI: 11.9–13.9), and the cardiovascular mortality rate was 5.7% (95% CI: 5.1–6.4). The leading cause of cardiovascular death was ischemic heart disease (290 deaths, 57.3%), followed by cerebrovascular disease (84 deaths, 16.6%). Factors significantly associated with all-cause mortality included older age, diabetes mellitus, glomerular filtration rate, HDL-cholesterol, left ventricular ejection fraction, left atrial and left ventricular diastolic dimensions, urgent hospitalization, on-pump surgery, and length of hospital stay.

**Conclusion:**

Patients undergoing CABG at a tertiary care center exhibited moderate 5-year all-cause and cardiovascular mortality rates. These findings highlight key prognostic factors that may support risk stratification and guide postoperative management strategies.

## Introduction

Coronary artery disease (CAD) remains the leading cause of morbidity and mortality worldwide, particularly among individuals over 30 years of age.^1^ Although multinational registries highlight notable differences in clinical profiles and outcomes, Central and South American countries consistently report higher rates of Diabetes Mellitus (DM), hypertension, myocardial infarction, and cardiovascular mortality.^2-4^ Despite this significant disease burden, these regions are often underrepresented in large-scale studies.

In Brazil, cardiovascular diseases account to one-third of all deaths, with CAD responsible for early half of these fatalities.^5^ Although initiatives led by national cardiology and cardiovascular surgery societies aim to improve care, there remains a scarcity of data on long-term outcomes for these patients.^6-8^

This study aims to address this gap by assessing five-year all-cause and cause-specific mortality in a cohort of coronary artery bypass graft (CABG) survivors. Additionally, we seek to identify key risk factors associated with these outcomes.

## Methods

This study was approved by the hospital’s Ethics Committee (protocol no. 4.513.250), which waived the requirement for individual patient consent. Due to legal constraints, the data cannot be publicly shared. The study adheres to the Strengthening the Reporting of Observational Studies in Epidemiology (STROBE) guidelines.^9^

### Patients and setting

This retrospective cohort study was conducted at the Instituto do Coração (Faculty of Medicine, University of São Paulo), Brazil. Patients who underwent coronary artery bypass grafting (CABG) between 2007 and 2018 and survived the initial hospitalization were included. Only the first CABG procedure was considered for inclusion. Patients residing outside São Paulo State, those younger than 18 years, individuals who underwent combined valvular or aortic procedures, and those who died during the qualifying hospitalization were excluded.

### Data sources and definitions

Clinical data were collected from electronic health records, including laboratory tests, echocardiography, medication use, length of hospitalization, and surgical details. The estimated glomerular filtration rate (eGFR) was calculated according to the 2021 Chronic Kidney Disease Epidemiology Collaboration method, and chronic kidney disease was defined as eGFR ≤ 60 mL/min/1.73 m^2^.^10^ DM was defined as the use of antidiabetic medication, glycated hemoglobin > 6.5%, or fasting glucose > 126 mg/dL.^1 1^ Hypertension was defined as the prescription of angiotensin-converting enzyme inhibitors, angiotensin receptor blockers, calcium channel blockers, clonidine, or diuretics.

Mortality data, including the cause of death as codified by the International Classification of Diseases, were retrieved from Fundação Sistema Estadual de Analise de Dados, a governmental agency database. This agency accounts for the civil registry of the state of São Paulo.^12^ As no information was available for patients residing in other states, they were excluded from this report.

Data from electronic health records and governmental mortality databases were linked using patient identifiers such as name, mother’s name, identity number, and date of birth, according to pre-specified matching criteria. These criteria included both exact and partial matches. All matched pairs (one record from each database) were validated by two independent investigators. A pair was considered valid if both investigators agreed; discrepancies were resolved by consensus. Data confidentiality was ensured by performing the linkage on-site at the governmental agency, with only the two investigators having access to identified records.

### Follow-up and outcomes

Follow-up started on the day of hospital discharge and finished upon death, as recorded in the death certificates. Patients without a death certificate were assumed to be alive, and their follow-up was censored at the last contact with the hospital (such as clinic visit, blood testing or echocardiography), or on December 31, 2018. As a major tertiary center in Brazil, our institution provides ongoing care for most surgical patients. Cardiovascular mortality was defined as International Classification of Diseases chapter I codes.

### Statistical analysis

Categorical variables were presented as absolute numbers and percentages, while continuous variables were expressed as medians with Interquartile Ranges (IQRs). Continuous variables were assessed for normality using graphical analysis and the Kolmogorov–Smirnov test. Given the retrospective nature of this study, no sample size calculations were performed; instead, all eligible participants were included. Baseline comparisons with excluded participants were conducted using the Wilcoxon rank-sum test and the chi-squared test, as appropriate. All-cause mortality was estimated using the Kaplan–Meier method, whereas cause-specific mortality was assessed using the cumulative incidence approach.

Risk factors were evaluated using a Cox proportional hazards model. Initially, all variables were eligible for inclusion. Variables identified as highly correlated – based on subject-matter expertise, Spearman’s and Pearson’s correlations – were excluded. Categorical variables with very low or very high prevalence (<5% or >95%) were also excluded due to statistical power considerations. Continuous variables were assessed for non-linearity using Martingale residuals and graphical analysis of preliminary models. Non-linear variables were modeled with restricted cubic splines (knots at the 10th, 50th, and 90th percentiles). The proportional hazards assumption was tested using Schoenfeld residuals. All variables were first analyzed in unadjusted models and subsequently in fully adjusted models.

Statistical analyses were performed with R version 4.5.0.^1 3^ All analyses were considered exploratory, and no adjustments were made for multiple testing. A significance level of 5% was adopted. Results are reported as p-values, Hazard Ratios (HR), and 95% confidence intervals (95% CI).

### Handling of missing data

Mortality estimates were performed on a complete-case basis. For the risk factor analysis, data were assumed to be missing at random. Therefore, multiple imputation was conducted using the predictive mean matching algorithm with bootstrapping with replacement, as implemented by the aregImpute function of the Hmisc package (version 5.1-1).^14^

## Results

Initially, a total of 9,008 eligible patients who underwent CABG between 2007 and 2018 were identified. Of these, 2,075 patients were excluded due to various reasons (Figure 1), and 6,933 patients were included in the analysis. Of those, 1,075 death certificates retrieved.

**Figure 1:** – Study flowchart. *Patients could meet more than one criteria.

Compared to those excluded, the included population had a higher prevalence of men (72% vs 68%, p < 0.001), a lower prevalence of chronic kidney disease (29 vs 39%, p < 0.002), was comprised of younger patients (median age 63 vs 67 years, p < 0.001), and had higher median left ventricular ejection fraction (60 vs 57%, p < 0.001). Other sample characteristics are reported in Table 1. The total follow-up period was 31,610 patient-years (median 4.0 years, IQR 1.4-7.3).

**Table 1:** – Baseline clinical, echocardiographic and surgical characteristics of the patients (n=6,933)

	**Entire sample (N = 6933)**	**Death during follow up**		**Missing data**
		**No (N = 5858)**	**Yes (N = 1075)**	
**Men, n (%)**	4,854 (72)	4,142 (73)	712 (69)	237 (3.4%)
**Age, years, Median (IQR)**	63 (57-70)	63 (56-69)	67 (59-73)	0 (0%)
**Chronic kidney disease, n (%)**	1,918 (29)	1,465 (26)	453 (44)	237 (3.4%)
**Diabetes mellitus, n (%)**				**0 (0%)**
Insulin-dependent	1,440 (21)	1,149 (20)	291 (27)	
Non-insulin-dependent	1,544 (22)	1,310 (22)	234 (22)	
No DM	3,949 (57)	3,399 (58)	550 (51)	
**High blood pressure, n (%)**	4,742 (69)	3,912 (67)	830 (78)	24 (0.3%)
**Total cholesterol, mg/dL, Median (IQR)**	164 (137-197)	164 (137-197)	164 (136-196)	1,107 (16%)
**LDL-cholesterol, mg/dL, Median (IQR)**	95 (73-124)	95 (72-124)	97 (75-125)	1,318 (19%)
**HDL-cholesterol, mg/dL, Median (IQR)**	37 (31-44)	37 (31-44)	36 (31-44)	1,138 (16%)
**Triglycerides, mg/dL, Median (IQR)**	131 (94-185)	132 (95-186)	128 (91-182)	1,107 (16%)
**Creatinine, mg/dL, Median (IQR)**	1.07 (0.91-1.29)	1.06 (0.91-1.26)	1.17 (0.94-1.49)	0 (0%)
**Glomerular filtration rate, mL/min/1.73 m^2^, Median (IQR)**	73 (57-88)	74 (59-90)	64 (46-83)	237 (3.4%)
**LV ejection fraction, %, Median (IQR)**	60 (51-65)	61 (53-65)	57 (42-63)	2,046 (30%)
**LV diastolic diameter, mm, Median (IQR)**	50 (46-54)	50 (46-53)	51 (47-56)	2,155 (31%)
**Type of internal thoracic artery graft, n (%)**				1,091 (16%)
Double	680 (12)	643 (13)	37 (4,9)	
Simple	4,783 (82)	4,154 (82)	629 (83)	
No internal thoracic artery graft	379 (6,5)	287 (5,6)	92 (12)	
**Saphenous vein graft, n (%)**	5,221 (89)	4,531 (89)	690 (91)	1,091 (16%)
**Pattern of graft selection, n (%)**				**1,091 (16%)**
Arterial	621 (11)	553 (11)	68 (9,0)	
Mixed	4,852 (83)	4,252 (84)	600 (79)	
Venous	369 (6,3)	279 (5,5)	90 (12)	
**Grafted territories, n (%)**				**1,091 (16%)**
One	617 (11)	530 (10)	87 (11)	
Two	2,601 (45)	2,245 (44)	356 (47)	
Three	2,624 (45)	2,309 (45)	315 (42)	
**Graft to LAD, n (%)**	5,742 (98)	5,006 (98)	736 (97)	1,091 (16%)
**Graft to RCA, n (%)**	3,580 (61)	3,148 (62)	432 (57)	1,091 (16%)
**Graft to CXA, n (%)**	4,369 (75)	3,793 (75)	576 (76)	1,091 (16%)
**On-pump surgery, n (%)**	5,978 (86)	5,139 (88)	839 (78)	3 (<0.1%)
**Urgent hospital admission, n (%)**	3,144 (45)	2,594 (44)	550 (51)	3 (<0.1%)
**Hospital days before surgery, Median (IQR)**	5.7 (1.8-8.7)	5.5 (1.4-8.2)	7.0 (3.6-12.0)	56 (0.8%)
**Hospital days after surgery, Median (IQR)**	9 (7-13)	8 (7-12)	11 (8-19)	56 (0.8%)
**Surgery year, Median (IQR)**	2012 (2009-2015)	2013 (2010-2016)	2009 (2008-2012)	0 (0%)

LVEF: left ventricular ejection fraction; LV: Left Ventricle; LAD: left anterior descending artery; RCA: right coronary artery; LCX: left circumflex artery; on-pump surgery: surgery with cardiopulmonary bypass.

### All-cause and cause-specific mortality

During the study period, 1,075 deaths were recorded (Figure 2). The five-year all-cause mortality rate was 12.9% (95% CI 11.9-13.9), with cardiovascular deaths accounted for 5.7% (95% CI 5.1-6.4) of cases (Central Illustration). Cardiovascular disease was the leading cause of death, followed by malignant neoplasms and respiratory diseases (Table 2).

**Figure 2:** – Cumulative all-cause and cause-specific deaths.

**Table 2:** – All-cause and cause specific deaths, according to follow-up time

	**n**	**%**	**Incidence at year of follow-up, % (95CI)**		
**Cause of death**			**1**	**5**	**10**
All causes			2.7 (2.3-3.1)	12.9 (11.9-13.9)	33.7 (31.6-35.7)
Diseases of the circulatory system	506	47.1	1.6 (1.3-2.0)	5.7 (5.1-6.4)	15.6 (14.1-17.2)
Neoplasms	163	15.2	0.2 (0.11-0.35)	2.1 (1.75-2.61)	5.2 (4.36-6.18)
Diseases of the respiratory system	108	10.0	0.29 (0.18-0.45)	1.23 (0.95-1.59)	3.50 (2.81-4.36)
Others	298	27.7	0.57 (0.41-0.79)	3.85 (3.32-4.46)	9.42 (8.29-10.71)

More than half (290 cases, 57.3%) of cardiovascular deaths were attributed to CAD, followed by cerebrovascular disease (84 cases, 16.6%). Malignant neoplasms were primarily of the digestive system (57 cases, 35%), respiratory system (31 cases, 19%) and genital or breast neoplasms (26 cases, 16%).

### Associated risk factors for mortality

Adjusted and unadjusted analyses of risk factors associated with all-cause mortality are presented in Figure 3. After multivariable adjustment, age, diabetes (particularly insulin-dependent), HDL-cholesterol, eGFR, left ventricular ejection fraction, left atrial enlargement, left ventricular diastolic dimension, on-pump surgery, urgent hospital admission, and both pre- and post-procedural hospitalization days were all independently associated with mortality.

**Figure 3:** – Associated factors with all-cause mortality.

eGFR showed a non-linear association with the outcome: individuals with an eGFR of 57 mL/min/1.73 m^2^ (3rd quartile), compared with those at 88 mL/min/1.73 m^2^ (1st quartile), had an HR of 1.20 (95% CI 1.08–1.34, p < 0.01). The risk remained stable above 60 mL/min/1.73 m^2^ but increased substantially below this threshold (Figure 4).

**Figure 4:** – Non-linear association between (A) estimated glomerular filtration rate, and (B) hospitalization days after surgery, and risk of death.

Similarly, post-procedural hospitalization days demonstrated a non-linear relationship with mortality. Risk increased rapidly and reached a plateau around the 16th post-procedure day (Figure 4). Compared with the 1st quartile (7 days), individuals in the 3rd quartile (13 days) had an HR of 1.38 (95% CI 1.20–1.59, p < 0.01).

Cardiovascular death was associated with largely the same risk factors, with a few exceptions (Figure 5). HDL-cholesterol and pre-procedure hospitalization days were not associated with the outcome (both p > 0.05). In contrast, male sex (HR 0.77, 95% CI 0.62–0.95, p = 0.02), exclusive arterial graft-only revascularization (HR 0.55 vs. mixed graft types, 95% CI 0.38–0.79, p < 0.01), and hypertension (HR 1.44, 95% CI 1.00–2.08, p = 0.05) were significantly associated with cardiovascular mortality.

**Figure 5:** – Associated factors with cardiovascular death.

## Discussion

### Main findings

In this cohort of CABG survivors, the five-year all-cause mortality rate was 12.9% (95% CI 11.9–13.9), with cardiovascular disease being the predominant cause of death, accounting for 5.7% (95% CI 5.1–6.4) of cases. Notably, CAD itself was responsible for 57.3% of these cardiovascular deaths. Several factors—including age, diabetes, higher HDL-cholesterol, lower eGFR, increased left atrial and ventricular dimensions, lower left ventricular ejection fraction, off-pump surgery, urgent hospitalization, and prolonged hospital stay—were associated with increased mortality. Male sex, arterial-only graft revascularization, and hypertension were specifically associated with cardiovascular mortality.

### Prognosis

Although this is a single-center study, our cohort is comparable to the Brazilian Society of Cardiovascular Surgery registry, a multicenter study conducted across 17 institutions in four of the five Brazilian geographic regions. In that registry, 2,929 patients were included, with a median age of 63.5 years; 29% were women; diabetes was present in 42.5%, and hypertension in 84.5%.^6^ Unfortunately, long-term follow-up data from that cohort have not been published.

Overall, we found mortality rates consistent with those reported in the international literature. For example, the New York State Registry, including 8,597 patients, reported a 5-year mortality rate of 17.6%, whereas data from the STS Adult Cardiac Surgery Database showed mortality rates of 8.1% at one year and 23.3% at three years.^15,16^ An Icelandic registry documented a 5-year mortality rate of 10.1%, including surgical events.^17^ Furthermore, randomized clinical trials have reported 5-year mortality rates ranging from 10% to 15% in CABG groups, although these figures include perioperative mortality.^1^

Considering that international guidelines define high cardiovascular risk as an annual cardiovascular mortality rate greater than 3%, our population would be classified as having moderate risk.^18-24^

### Associated risk factors

Age, diabetes, and eGFR were consistently associated with both all-cause and cardiovascular mortality, as expected. Intriguingly, eGFR and postoperative hospitalization days demonstrated a non-linear relationship with risk. While mortality risk did not change substantially for eGFR values above 60 mL/min/1.73 m^2^, it increased markedly below this threshold. This finding aligns with previous studies in post-CABG and general population cohorts.^25,26^

Despite traditionally being linked to a favorable prognosis, HDL-cholesterol was associated with all-cause mortality (but not with cardiovascular death). Although this is likely a type I error (false positive), it is noteworthy that a large population-based study reported a U-shaped relationship between HDL-cholesterol and mortality.^27^

Echocardiographic markers of structural myocardial damage, such as left ventricular dimension and ejection fraction, were predictably associated with both all-cause and cardiovascular mortality.^28^ Additionally, left atrial enlargement was also associated with mortality. Because heart failure – regardless of ejection fraction – is closely linked to CAD, left atrial enlargement may reflect more extensive disease and elevated filling pressures.^29,30^ Moreover, left atrial enlargement is related to atrial fibrillation, and incident atrial fibrillation has been shown to be associated with stroke and mortality after surgery.^3 1^

Our findings suggest that the type of revascularization (total arterial, total venous, or mixed) was associated with cardiovascular mortality, but not with all-cause mortality. Randomized trials have shown somewhat inconsistent results: while bilateral internal thoracic artery grafts have not demonstrated superiority over single internal thoracic artery grafts, radial artery grafts have been associated with better outcomes compared with saphenous vein grafts.^32,33^ Observational studies, in general, indicate a benefit of total arterial revascularization.^34,35^

The effectiveness of on-pump versus off-pump surgery has been extensively debated. Non-randomized studies, including ours, suggest potential long-term survival benefits with on-pump procedures; however, these findings have not been consistently replicated in randomized trials.^36^

### Limitations

This study has several limitations. First, all analyses were exploratory in nature and should be interpreted primarily as hypothesis-generating. Second, the use of secondary data limited access to certain key variables, such as coronary anatomy, myocardial ischemia, symptoms, and quality-of-life measures. Third, our access to death certificates was restricted to the State of São Paulo, which may have resulted in the exclusion of deaths occurring outside the region. To mitigate this issue, we included only patients residing in São Paulo and actively followed at the Instituto do Coração.

## Conclusions

Patients who underwent CABG in this Brazilian cohort experienced moderate five-year mortality rates according to international standards, with nearly half of these deaths attributable to cardiovascular causes. The main risk factors associated with mortality included age, diabetes mellitus, eGFR, off-pump surgery, structural cardiac abnormalities, urgency of hospitalization, and length of hospital stay.
